# The impact of *sseK2* deletion on *Salmonella enterica* serovar typhimurium virulence in vivo and in vitro

**DOI:** 10.1186/s12866-019-1543-2

**Published:** 2019-08-07

**Authors:** Xiaojie Zhang, Lei He, Chunjie Zhang, Chuan Yu, Yadong Yang, Yanyan Jia, Xiangchao Cheng, Yinju Li, Chengshui Liao, Jing Li, Zuhua Yu, Fuyu Du

**Affiliations:** 1 The Key Lab of Animal Disease and Public Health, Henan University of Science and Technology, 263 Kaiyuan Avenue, Luoyang, 471023 Henan China; 2 Luoyang Key Laboratory of Live Carrier Biomaterial and Animal Disease Prevention and Control, Luoyang, 471023 Henan China; 3 Luoyang Polytechnic, 6 Airport Road, Luoyang, 471023 Henan China

**Keywords:** *Salmonella* typhimurium, Δ*sseK2* mutant, Biofilm formation, Virulence

## Abstract

**Background:**

*Salmonella enterica* is regarded as a major public health threat worldwide. *Salmonella* secretes the novel translocated effector protein K2 (SseK2), but it is unclear whether this protein plays a significant role in *Salmonella enterica* Typhimurium virulence.

**Results:**

A Δ*sseK2* mutant of *S.* Typhimurium exhibited similar growth curves, adhesion and invasive ability compared with wild-type (WT) bacteria. However, deletion of *sseK2* rendered *Salmonella* deficient in biofilm formation and the early proliferative capacity of the Δ*sseK2* mutant was significantly lower than that of the WT strain. In vivo, the LD_50_ (median lethal dose) of the Δ*sseK2* mutant strain was increased 1.62 × 10^3^-fold compared with the WT strain. In addition, vaccinating mice with the Δ*sseK2* mutant protected them against challenge with a lethal dose of the WT strain. The ability of the Δ*sseK2* mutant strain to induce systemic infection was highly attenuated compared with the WT strain, and the bacterial load in the animals’ internal organs was lower when they were infected with the Δ*sseK2* mutant strain than when they were infected with the WT strain.

**Conclusions:**

We conclude that *sseK2* is a virulence-associated gene that plays a vital role in *Salmonella* virulence.

**Electronic supplementary material:**

The online version of this article (10.1186/s12866-019-1543-2) contains supplementary material, which is available to authorized users.

## Background

*Salmonella enterica* is a facultative intracellular Gram-negative pathogen that has a wide range of hosts and is regarded as a major public health concern worldwide [1]. Various *Salmonella* and serovars still pose a critical threat to human health, especially in developing countries [2, 3]. *Salmonella enterica* can cause a variety of animal diseases, such as typhoid fever. *Salmonella* pathogenic is mainly facilitated by a type III secretory system (T3SS) encoded by the genes in *Salmonella* pathogenicity islands 1 and 2 (SPI-1 and SPI-2) [4, 5]. SPI-1 is mainly expressed in the intestines to mediate invasion of epithelial cells by *Salmonella*, while SPI-2 can facilitate intracellular proliferation of *Salmonella* in the host’s macrophages [6, 7]. Although *sseK2* is an important gene that is located on SPI-2 [8], it is unknown whether this gene plays a role in *Salmonella* virulence.

The SseK proteins in *S.* Typhimurium are regarded as T3SS effectors, and include SseK2 (STM2137), SseK1 (STM4157) and SseK3 (sb26) [9]. Interestingly, these proteins are highly similar in different bacterial species, such as the enterohemorrhagic *Escherichia coli* and *Citrobacter rodentium* [10, 11]. The SseK1 and SseK2 proteins are encoded by genes located in the islands on bacterial chromosomes, and share 61% identity at the amino acid level [10]. Both SseK1 and SseK3 are found to inhibit the activation of the proinflammatory transcription factor NF-κB and work as GlcNAc (*N*-acetylglucosamine) transferases that could modify the TNFR1-associated death domain protein TRADD [12, 13]. Notably, all of the key residues necessary for SseK3 enzyme activity are conserved in SseK2 [12]. While a previous study that deletion of the *sseK1* gene can significantly reduce virulence [14], there is no evidence regarding how the presence of the *sseK2* gene promotes bacterial virulence. The *Salmonella* protein K2, which is a novel translocated protein, is a secreted T3SS effector protein that is involved in bacterial translocation. In addition, the gene encoding SseK2 is highly conserved in the *Salmonella* genome [10]. Research has suggested that SseK2 is also an GlcNAc transferase [15], but it is uncertain whether *sseK2* has an effect on *S.* Typhimurium virulence. Gaining a better understanding of the effects of *sseK2* on bacterial virulence may help improve the design of live attenuated vaccines, which are regarded as an effective means of preventing *Salmonella* infection [16, 17].

In this study, we implemented a SacB/sucrose counterselection strategy to obtain a Δ*sseK2* mutant. The potential virulence of Δ*sseK2* mutant was examined in both in vitro and in vivo models of infection. We verified that deletion of *sseK2* reduces *Salmonella* virulence, indicating that *sseK2* is a *Salmonella* virulence-associated gene. Our data clearly showed that *sseK2* plays a vital role in *Salmonella* virulence.

## Results

### Analysis of **the** Δ*sseK2* mutant

**Fig. 1:**
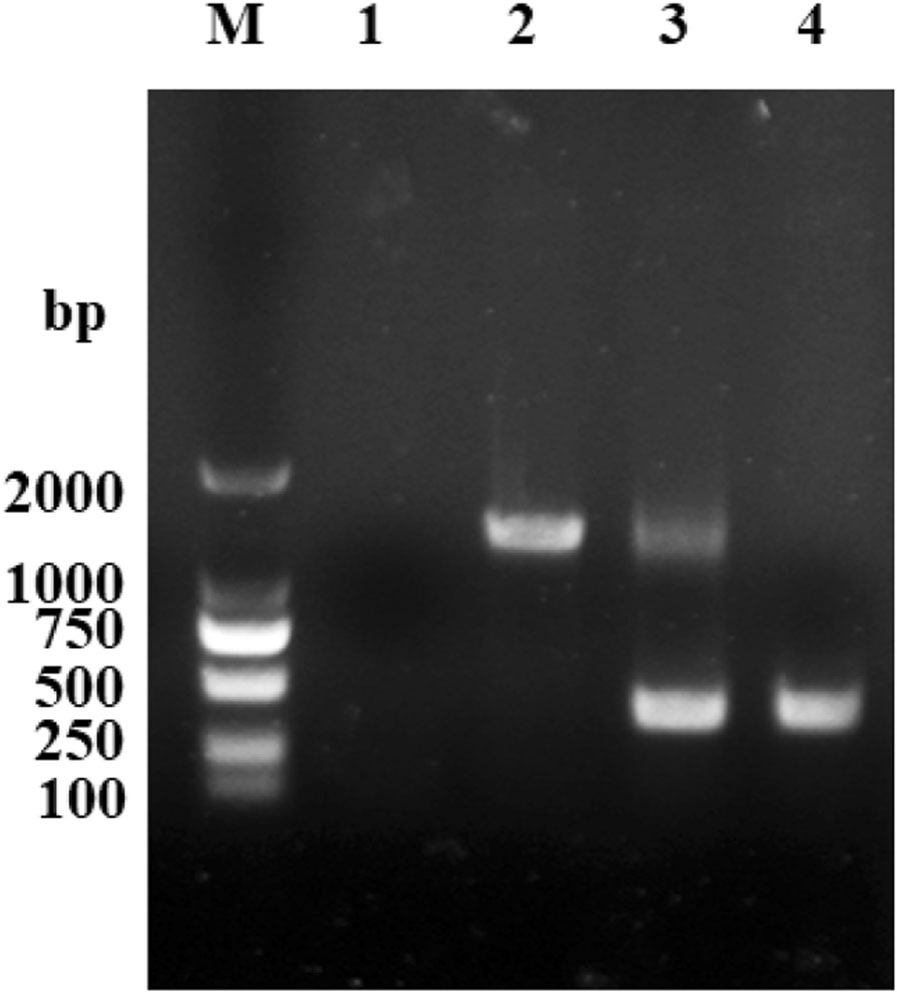
Identification of the Δ*sseK2* mutant by PCR (polymerase chain reaction). M: marker (DL2000); 1: negative control; 2: WT; 3: single-crossover Δ*sseK2* mutant; 4: double-crossover Δ*sseK2* mutant

An in-frame deletion of the *sseK2* gene was cloned into pRE112 to create a recombinant suicide plasmid. The Δ*sseK2* mutant was PCR (polymerase chain reaction)-amplified using primers *sseK2* - F and *sseK2* - R. We obtained an approximately 459-bp fragment (Fig. 1). The *sseK2* deletion was confirmed by DNA sequencing.

### Growth characteristics of the Δ*sseK2* mutant

**Fig. 2:**
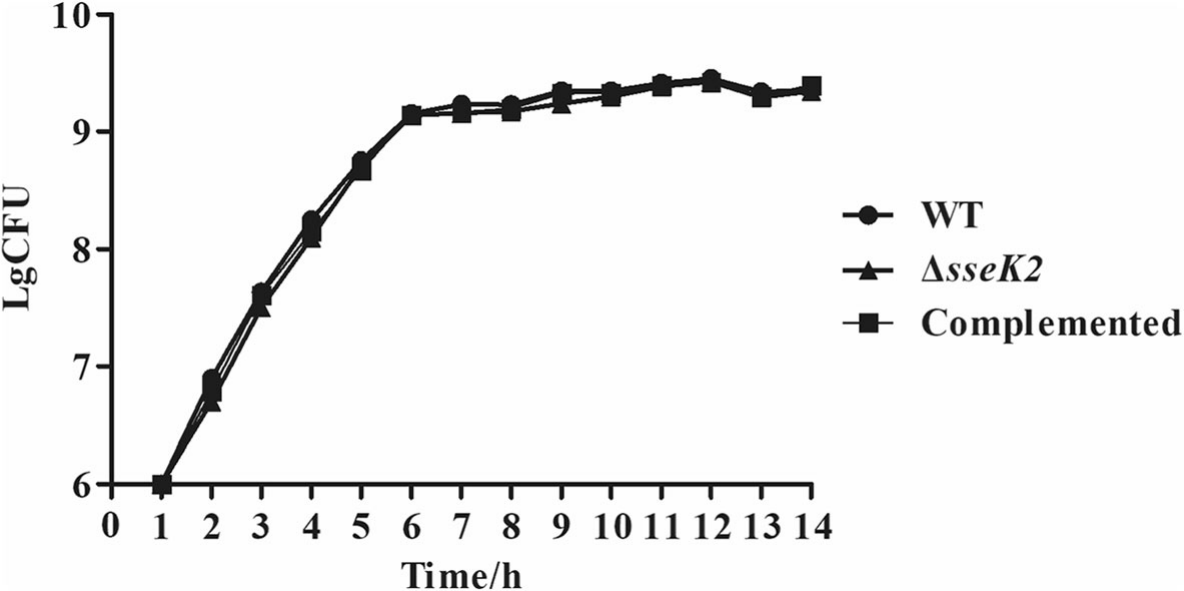
Growth curves for the WT, Δ*sseK2*, and complemented strains. All strains were cultured in LB medium. Growth curves were generated by determining viable cell counts

The Δ*sseK2* mutant, the WT strain, and a complemented strain were verified by antibiotic selection and PCR, and the results showed that their growth characteristics in LB (LuriaBertani) medium did not differ greatly (Fig. 2). This indicated that *sseK2* deletion did not influence the growth characteristics of *S.* Typhimurium.

### Stability of the Δ*sseK2* mutant

**Fig. 3:**
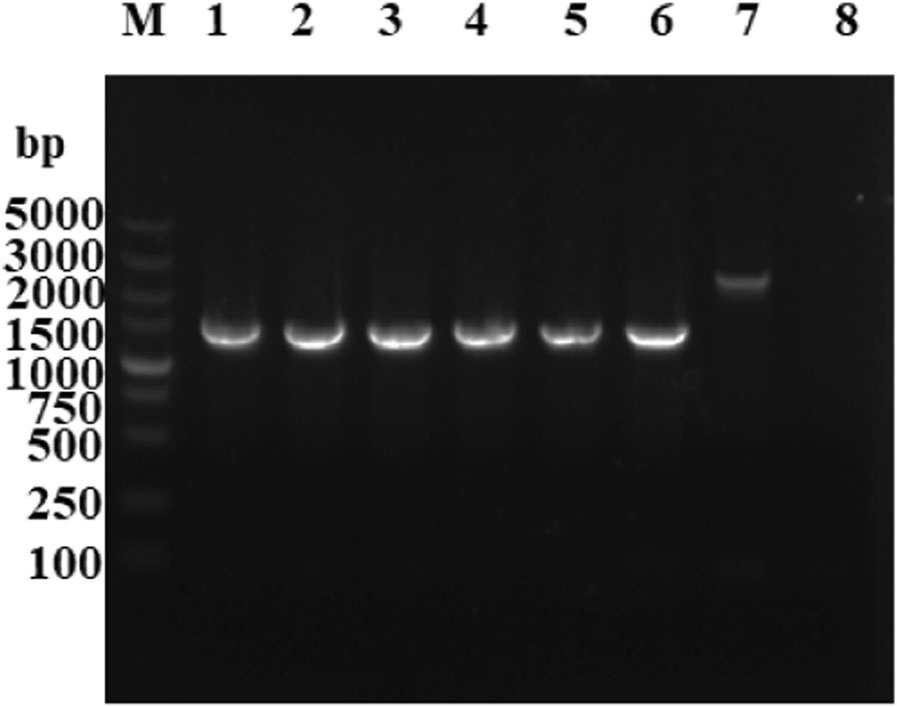
PCR identification of Δ*sseK2* mutant stability. M: marker (DL5000); 1–6: PCR- amplified products from the 10th, 20th, 30th, 40th, 50th and 60th passages of the Δ*sseK2* mutant; 7: wild-type *Salmonella* Typhimurium SL1344; 8: negative control

The Δ*sse*K2 mutant was serially passaged 60 times in LB medium, and the presence of the *sseK2* deletion was then assessed by PCR (Fig. 3). The *sseK2* deletion was still detectable in the Δ*sseK2* mutant strain, indicating that this strain has good genetic stability.

### Biofilm formation and morphology assay

**Fig. 4:**
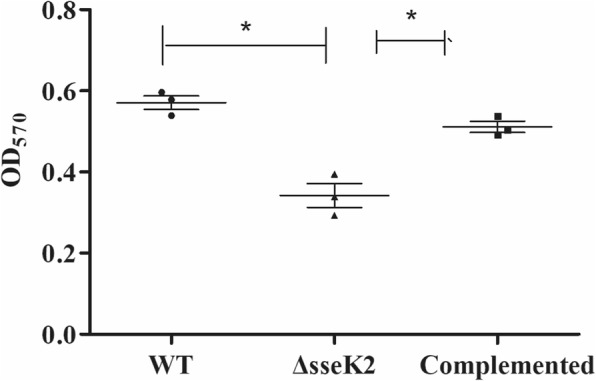
Biofilm formation by the WT, Δ*sseK2*, and complemented strains. The asterisk (*) indicates statistically significant differences between the WT, Δ*sseK2* and its complemented strains (*P* < 0.05). OD_570_ values for the WT, Δ*sseK2* and complemented strains as determined by crystal violet staining

Biofilm formation is an important aspect of *Salmonella* virulence [18–20]. Therefore, we assessed biofilm formation in the three strains noted above. The results showed that the ability of the Δ*sseK2* mutant to form biofilm was significantly lower than that of the WT and complemented strains, based on OD_570_ values (Fig. 4), indicating that *sseK2* is necessary for the formation of biofilm by *S.* Typhimurium*.*

### The Δ*sseK2* mutant has altered biological activity in vitro

**Fig. 5:**
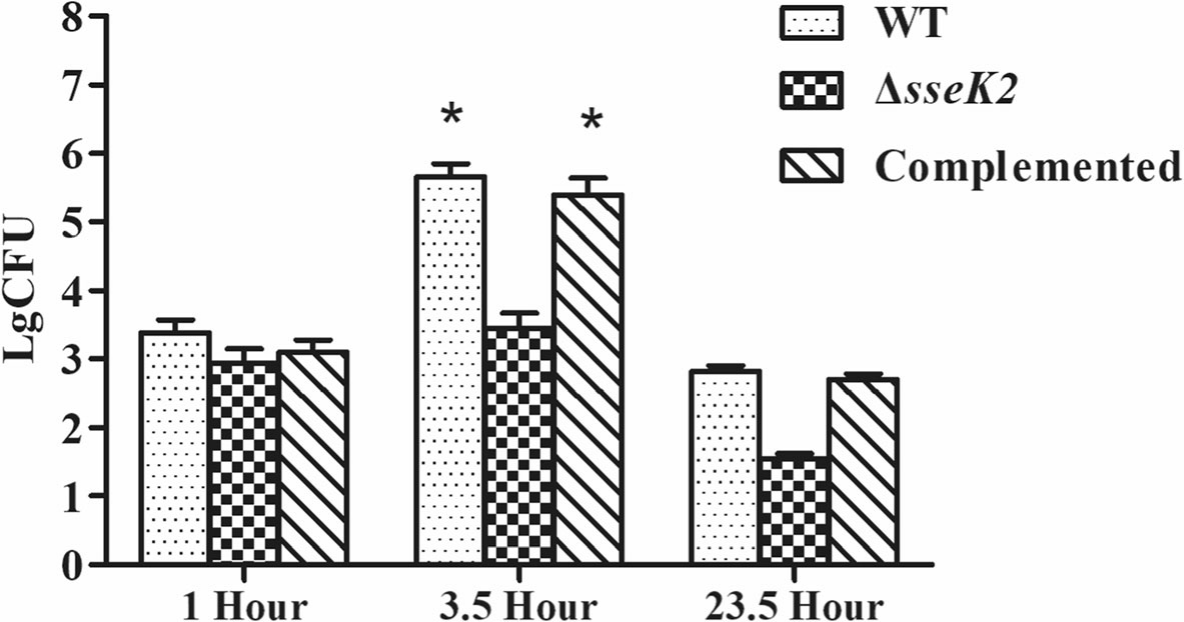
Intracellular proliferation of the WT, Δ*sseK2*, and its complemented strains in J774A.1. cells. The WT, Δ*sseK2*, and complemented strains were co-incubated with J774A.1. cells, and the number of bacteria was counted at 1, 3.5 and 23.5 h. The asterisk (*) indicates that there was a statistically significant difference between the WT, Δ*sseK2* and complemented strains at 3.5 h compared with 1 h. (*P* < 0.05)

The invasive and adhesive abilities of the *sseK2* mutant and the complemented strain were similar to those of the WT strain (Table 1). This suggests that *sseK2* does not play an important role in promoting *S.* Typhimurium attachment to and invasion of host macrophages. The Δ*sseK2* mutant intracellular load increased by 0.5 log over a period of 3.5 h (Fig. 5). In contrast, the intracellular load of the WT and complemented strains increased by 2.3 logs over the same time period. However, over a 23.5 h period, the number of Δ*sseK2* mutant bacteria decreased 1.9 logs, whereas the number of WT bacteria decreased by 2.8 logs. These data show that there is a significant difference in intracellular proliferation between the Δ*sseK2* mutant and the WT strain (*P* < 0.05), indicating that SseK2 is required for intracellular survival of *Salmonella* in vitro.

**Table 1:** Role of the Δ*sseK2*, WT and complemented strains in adherence to and invasion of J774A.1 cells

Strains	Percentage adherence (no.adhered/no. inoculated)	Percentage invasion (no.invaded/no. inoculated)
Δ*sseK2*	1.63 ± 0.16	2.96 ± 0.12
WT	1.8 ± 0.12	3.40 ± 0.41
Complemented	1.75 ± 0.13	3.1 ± 0.38

Values are mean ± SD

### The virulence characteristics of the Δ*sseK2* mutant in vivo

**Fig. 6:**
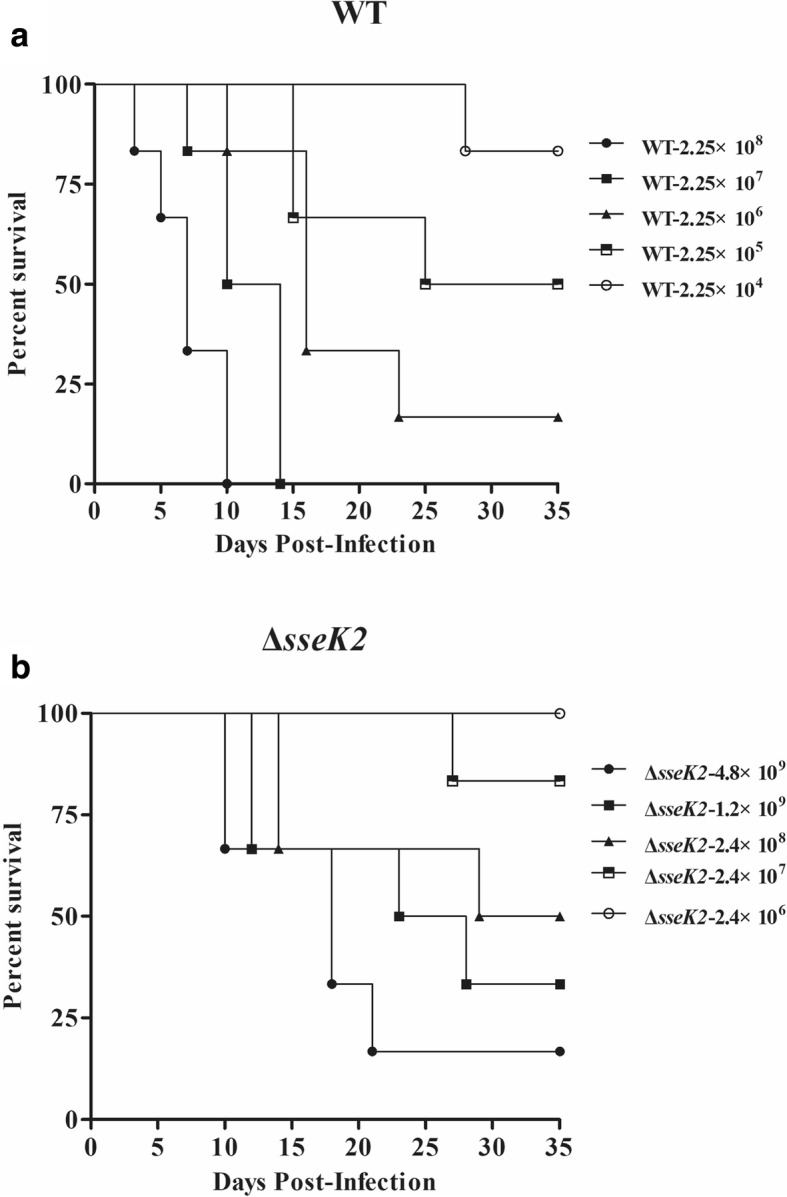
Percent survival of mice infected with the WT (**a**) or Δ*sseK2* mutant strain (**b**). The mice were inoculated by intraperitoneal injection, and mortality was monitored over 5 weeks

A mouse model of infection was established to determine whether *sseK2* deletion would alter the virulence characteristics of *S.* Typhimurium in vivo. The results from this experiment are shown in Fig. 6. All of the mice injected with 2.4 × 10^6^ CFU (colony-forming unit) of the Δ*sseK2* mutant survived, while the 16.7, 50, 33.3 and 83.3% of mice injected with 2.4 × 10^7^, 2.4 × 10^8^, 1.2 × 10^9^ or 4.8 × 10^9^ CFU, respectively, died (Fig. 6b). In contrast, 16.7, 50% or 83.3% of mice injected with 2.25 × 10^6^, 2.25 × 10^5^ or 2.25 × 10^4^ CFU, respectively, died (Fig. 6a). The LD_50_ values for the Δ*sseK2* mutant, the WT strain, and the complemented strain were 3.44 × 10^8^, 2.12 × 10^5^ and 4.30 × 10^5^ CFU, respectively. All of the mice in negative control group survived (data not shown).

**Fig. 7:**
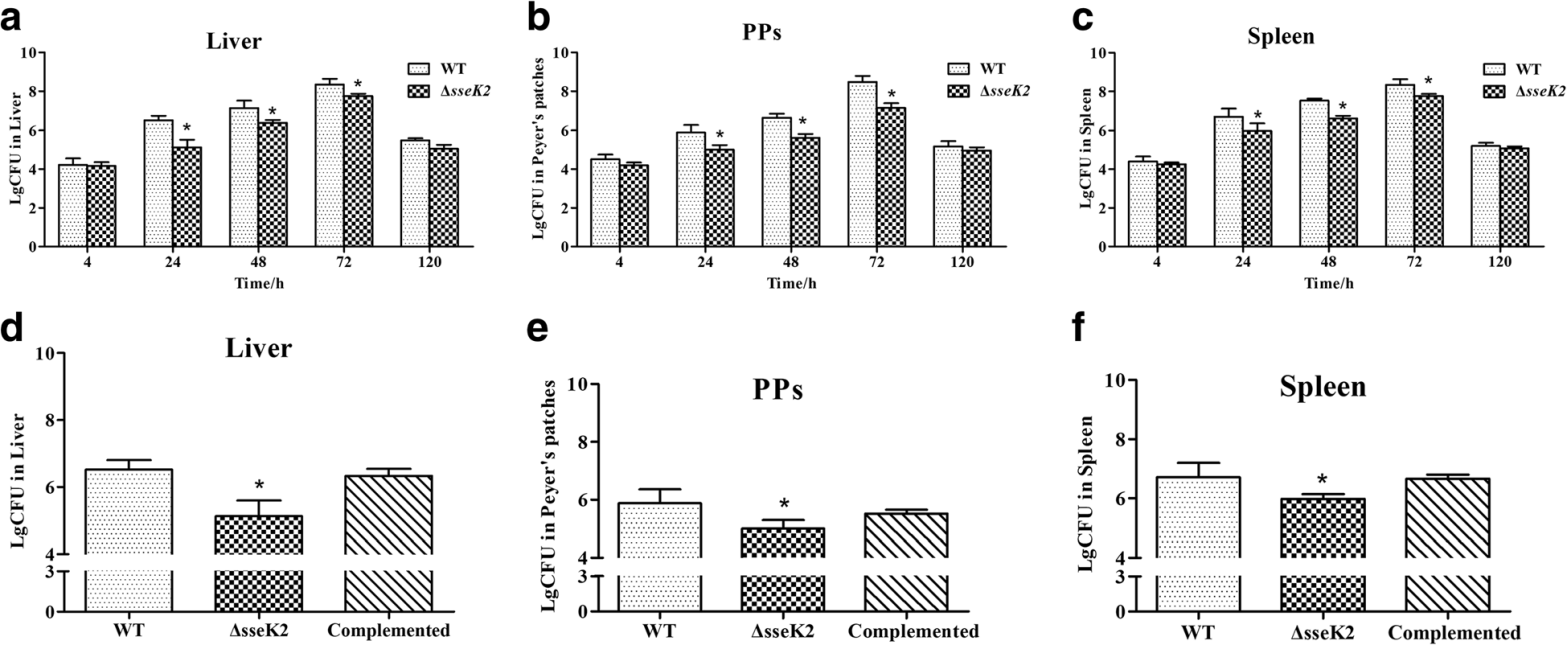
Analysis of WT and Δ*sseK2* mutant bacterial loads in the liver (**a**), PPs (**b**), and spleen (**c**) of mice. The asterisk (*) indicates statistically significant differences between the WT and Δ*sseK2* mutant strains (*P* < 0.05). The virulence of the complemented strain was evaluated based on the bacterial load in the liver (**d**), PPs (**e**), and spleen (**f**) of mice at 24 h

The number of Δ*sseK2* or WT bacteria in the mouse livers increased to 7.75 logs and 8.35 logs, respectively, between 4 h and 72 h and then decreased to 5.06 logs and 5.48 logs, respectively, at 120 h post-infection. This indicates that the bacterial load of the Δ*sseK2* mutant in the liver had begun to decrease. (Fig. 7a). In the Peyer’s patches (PPs), the Δ*sseK2* mutant and WT loads increased to 7.13 logs and 8.48 logs, respectively, at 4–72 h, but decreased to 4.96 logs and 5.17 logs, respectively, at 120 h (Fig. 7b). Meanwhile, the Δ*sseK2* mutant and WT loads in the spleen increased to 7.76 logs and 7.98 logs, respectively, between 4 h and 72 h post-infection. However, they decreased to 5.07 logs and 5.20 logs, respectively, after 120 h post-infection (Fig. 7c).

After 4 h of infection, *S.* Typhimurium could be recovered form in the liver, spleen, and PPs. At 24 h post-infection, there was a significant difference in the number of Δ*sseK2* mutant and WT bacteria recovered from the spleen, liver, and PPs. Moreover, in mice infected with the complemented strain, the number of bacteria was restored to the same level as in mice infected with the WT strain (Fig. 7d, e, f). The bacterial counts from in the spleen and liver were significantly lower than those from mice infected with the parental strain 24 h, 48 h, and 72 h post-infection (*P* < 0.05), but the counts for the complemented strain were restored to level of the parent strain.

## Discussion

The results from this study provide evidence that deletion of *sseK2* can significantly reduce the virulence of *Salmonella*. The *sseK2* deletion significantly decreased biofilm formation, the proliferation in macrophages in vitro, and the bacterial load in liver and spleen decreased in vivo infection models, indicating *sseK2* is a virulence-associated gene that plays a vital role in *Salmonella* virulence.

SseK2 is a novel translocated *S.* Typhimurium protein that is highly conserved among *Salmonella* strains [10]. However, the function of SseK2 was unknown until now. Biofilm formation is required for *Salmonella* to spread [21, 22]. *Salmonella* in biofilms are more resistant to hostile environments than individual bacteria [23, 24]. In vivo*,* biofilm formation helps bacteria evade the immune system and resist antibiotics-mediated killings, thereby resulting in chronic infection [23, 24]. Biofilm formation also contributes to the spread of *Salmonella* in vitro, because bacteria in biofilms are more resistant to disinfectants and physical stress than individual bacteria [25–27]. The biofilm matrix is mainly composed of curli fimbriae and cellulose [28]. A few proteinaceous compounds such as BapA and DksA have been reported to participate in biofilm formation [28]. We found that the ability of the Δ*sseK2* mutant to form biofilms was significantly decreased compared to the WT strain, indicating that the SseK2 protein could be a novel proteinaceous compound in biofilms. Furthermore, the ability to form biofilms is an important virulence factor during *Salmonella* infection. A strain of *Salmonella* that produces biofilm was more virulent in chickens than s non- biofilm-forming strain [29]. Thus, a decreased ability to form biofilms could be involved in the decreased virulence of Δ*sseK2* mutant. However, its influence on the biofilm formation in vivo should be explored in the future.

However, we did not observe any statistically significant difference in adhesion and invasion between the Δ*sseK2* mutant and the WT strain, indicating that, at least in vitro, S*almonella* invasion and adhesion are not controlled by *sseK2*. The apparent discrepancy between the effect of *sseK2* deletion on biofilm formation and its lack of effect on invasion and adhesion may be because *sseK2* is located in SPI-2 [8]. A major function of SPI-2 is to enable intracellular bacterial replication, while the principal role of the SPI-1 encoded secretion system is to facilitate bacterial invasion of epithelial cells [30]. Proliferation of the Δ*sseK2* mutant in host cells was significantly reduced by 10^− 2^-fold compared with the WT strain (Fig. 5). Thus, *sseK2* is necessary for biofilm formation and intracellular proliferation in vitro, but is dispensable for host cell adhesion and invasion. Interestingly, another protein, DksA, also plays an important role in biofilm formation but is dispensable for adhesion [31].

The LD_50_ of the Δ*sseK2* mutant strain was markedly increased compared with the WT strain. Mice were infected with WT strain, began to die on the third day post-infection, whereas mice infected with the Δ*sseK2* strain began to die on the tenth day post-infection (Fig. 6). We further tested the Δ*sseK2* mutant and WT strains in BALB/c mice to determine differences in bacterial burdens post-infection. Consistent with our in vitro results, the LD_50_ assay showed that the Δ*sseK2* mutant developed a systemic infection more closely than WT strain, indicating that *sseK2* could affect the ability of *S.* Typhimurium to establish systemic infection in vivo. *Salmonella* invades multiple organs in mice, such as the liver, spleen, and PPs. The bacterial loads of the complemented strain were similar to those of the WT strain in multiple organs, while bacterial loads of Δ*sseK2* mutant were lower than WT in multiple organs. Furthermore, the Δ*sseK2* mutant caused less tissue damage to the liver, PPs and spleen (data not shown). These data clearly show that *sseK2* may be involved in facilitating bacterial infection and that *sseK2* gene contributes to *S.* Typhimurium virulence.

It is crucial for live attenuated *Salmonella* vaccines to effectively confer protection against wild-type *S.* Typhimurium [32]. Therefore, we also evaluated the protective efficacy of the Δ*sseK2* mutant against oral challenge with SL1344. Only 62.5% mice vaccinated with the Δ*sseK2* mutant survived, whereas the control group exhibited 100% mortality (data not shown). Although the Δ*sseK2* mutant does not confer enough of a protective effect to be used as a new attenuated *Salmonella* vaccines, deletion of the *sseK2* gene and other genes could be a good way to construct a live attenuated vaccine, as deletion of two or three virulence-related genes is regarded as a good approach for designing novel attenuated *Salmonella* vaccine [33–35]. In addition, genetic stability and no reversion to virulent strain are necessary conditions for a vaccine candidate [36, 37]. The Δ*sseK2* mutant grew similarly to the WT strain and had a good genetic stability over 60 passages, which will be advantageous in developing novel vaccine.

## Conclusions

In summary, we constructed a Δ*sseK2* mutant of *S.* Typhimurium, and found that lack of *sseK2* affects *S.* Typhimurium pathogenicity by decreasing its virulence both in vitro *and* in vivo. This study provides a new and effective candidate for developing attenuated *Salmonella* vaccines.

## Methods

### Animals, bacterial strains, plasmids and culture conditions

Specific pathogen-free (SPF) BALB/c mice (age, 5–6 weeks; body weight, 20 ± 2 g) were obtained from the experimental animal center of Henan University of Science and Technology (Luoyang, China). This study was carried out in accordance with the regulations established by the Chinese Ministry of Science and Technology. All animals were subjected to a clinical examination to assess their physical appearance and the normality of their behavior, and those presenting signs of disease were removed. The mice were anesthetized with 20% urethane (ethyl carbamate) solution by intraperitoneal injection. 5 min later, the anesthetized mice were sacrificed by cervical dislocation. Then the corresponding animal experiments were started. All animals were humanely handled. This work adheres to ARRIVE guidelines (Additional file 1). The study was approved by the Institutional Animal Care and Use Committee (IACUC) of the College of Animal Science and Technology, Henan University of Science and Technology (no. 201706001). All of the animal experiments were performed in our laboratory.

The bacterial strains and plasmids used in this study are listed in Table 2. Liquid bacterial cultures were maintained in LB broth. The J774A.1 Macrophage cell line (Resource Center, IBMS, CAMS/PUMC 3111C0001CCC000222) was obtained from the American Type Culture Collection (ATCC, Manassas, VA). The cells were grown and maintained in Dulbecco’s Modified Eagle Medium (Sigma, China) supplemented with 10% fetal bovine serum (FBS) (Gibco, China) at 37 °C with 5% CO_2_.

**Table 2:** Bacterial strains and plasmids used in this study

Strain or plasmid	Characteristics	Source or reference
Strains		
SL1344	*Serovar Typhimurium*, wild-type	[38]
SL1344Δ*sseK2*	*sseK2* deletion mutant	In this study
χ7213	χ7213, containing plasmid of pRE*sseK2*, Cm^r^	In this study
Plasmids		Laboratory stock
pBluescriptIISK (+)	Phagemid cloning vector, oriCOLE1 *oriF1(+) bla lacZa*	In this study
pBSK*sseK2*	pBluescriptIIKSt, *sseK2*	[39]
pRE112	pGP704 suicide plasmid, pir dependent, oriT, oriV, sacB, Cm^r^	In this study
pREΔ*sseK2*	pRE112 dervative containing *ssek2* fused in-frame, Cm^r^	Laboratory stock
pBR322	oriColE1, Amp^r^Tc^r^	In this study
pBR322-*sseK2*	pBR322 carrying the full *sseK2*gene (Amp^r^)	

### Construction of the Δ*sseK2* mutant and its complemented strain

The Δ*sseK2* mutant was constructed using methods described previously [40, 41]. The primers are shown in Table 3. The *sseK2* gene was amplified by PCR, and the recombinant plasmid pBSK-*sseK2* containing the *sseK2* gene was constructed. The *sseK2* gene was deleted in-frame, and then the recombinant plasmid pRE112Δ*sseK2* was constructed and transformed into *E. coli*χ7213 (λpir) for mobilization into the WT by conjugation. Single-crossover transconjugants (in which the recombinant plasmid pRE112Δ*sseK2* was integrated into the chromosome) were identified and screened on LB plates containing chloramphenicol. Ten percent sucrose was added to LB plate without NaCl and with chloramphenicol and the pRE112 suicide plasmid removed from the single-crossover transconjugants. Chloramphenicol-sensitive colonies were selected, and the *sseK2* deletion was screened for by PCR using primers *sseK2*-F and *sseK2*-R. Subsequently, DNA sequencing was performed to confirm whether *sseK2* was deleted. The *sseK2* gene was cloned into the pBR322 plasmid for complementation studies.

**Table 3:** Sequences of the primers used in this study

Primer	Primer sequences	Restriction site
Δ*sseK2*-up-F	5′-TCTAGAATAGAAGAGGCCCAAAGA-3’	*Xba* I
Δ*sseK2*-up-R	5′-GGATCCATTTTTACACGCTTAAATTA-3’	*Bam*H I
Δ*sseK2*-down-F	5′-CTCGAG TCATGATAGCCTTGTTTTAC −3’	*Xho* I
Δ*sseK2*-down-R	5′-GGTACC ACACGGCGCACTATTAGA-3’	*Kpn* I
*sseK2*- F	5′-ACCACACTAACCAAAGCGCA-3’	
*sseK2* -R	5′-GCAGAGAATAATGGACCACAT-3’	
pBR -*sseK2*-F	5′-GGATCCATGGCACGTTTTAATGCC-3’	B*amH* I
pBR -*sseK2*-R	5′-CTCGACTTACCTCCAAGAACTGGCAG-3’	*Sal* I

### Growth and phenotypic characterization assay

Growth and phenotypic characterization was performed as previously described [42]. Three colonies were picked and inoculated into 10 ml of fresh LB broth, then incubated overnight at 37 °C. The overnight culture was diluted 1:100 into fresh LB broth and incubated overnight at 37 °C with shaking. The overnight cultures were then, serially diluted and plated to LB medium for plate counts. The index generation time was calculated based on the growth rate from 1 to 14 h.

### Stability of the Δ*sseK2* mutant

To determine the stability of the Δ*sseK2* cassette post-chromosomal integration, a liquid culture of the Δ*sseK2* strain (1% inoculum) was serially passaged 60 times every 12 h. Every 10 passages, DNA extraction for detection of Δ*sseK2* by PCR.

### Biofilm formation and morphotype assay

The biofilm formation and morphotype assay was performed as previously described [43]. Briefly, suspension of the WT, Δ*sseK2*, and complemented strains were adjusted to the same concentration, and at 28 °C for 72 h in a 96-well cell culture plate without shaking in a humidified environment. Each well of the 96-well plate was slowly washed three times with PBS (phosphate buffered saline) (pH 7.0), and then allowed to dry at 37 °C for 30 min. Then, 100 μl of crystal violet (10 mg/ml; Sigma) was added to each well of the 96-well plate for 30 min. Subsequently, 100 μl of absolute ethanol was added to each well, and biofilm formation was measured by determining the OD (optical density) at 570 nm.

### Adherence and invasion assays

Approximately 1 × 10^5^ J774A.1 cells were added to each well of a 24-well plate. The WT, Δ*sseK2* and complementary strains were then added to the J774A.1 cells at a multiplicity of infection (MOI) of 100:1. After incubation for 2 h at 37 °C, each well was washed three times with PBS. The wells were then treated with 0.25% trypsin, serially diluted, and plated, and colony counts were performed to determine the number of adhesive cells. For the invasion assay, 100 μg/ml gentamicin was added to each well for 1 h at 37 °C, and the cells were then lysed with 0.1% Triton X-100. To calculated the invasion rate, serially diluted cell lysates were evenly plated onto SS (*Salmonella* Shigella) agar, and the number of CFUs was determined. The data shown are representative of at least three independent experiments for each strain, which were performed in triplicate.

### Intracellular proliferation assay

Approximately 1 × 10^5^ J774A.1 murine macrophage cells were added to each well of 6-well plates. Subsequently, the cells were infected and the invasion assay was performed as previously described. After incubating for 30 min, the cells were washed three times, 150 μl of fresh medium containing gentamicin (50 μg/ml) was added to the wells. Two hours later, the infected cells were lysed with 1 ml 0.1% Triton X-100 for 10 min. The number of CFUs was determined by plating the cell lysates on SS agar.

### LD_50_ and persistence assay

Ninety-six (SPF) BALB/c mice were divided randomly into five subgroups, each of which contained three male mice and three female mice. Based on the results from the preliminary study, mice in the Δ*sseK2* mutant group were inoculated orally with 0.2 ml of liquid culture containing of 4.8 × 10^9^, 1.2 × 10^9^, 2.4 × 10^8^, 2.4 × 10^7^or 2.4 × 10^6^ CFU, the mice in the WT group received 2.25 × 10^8^, 2.25 × 10^7^, 2.25 × 10^6^, 2.25 × 10^5^, or 2.25 × 10^4^ CFU, and the mice in the complemented group received 2.2 × 10^8^, 2.2 × 10^7^, 2.2 × 10^6^, 1.1 × 10^5^, or 1.1 × 10^4^ CFU. Six of the mice were inoculated orally with 0.2 ml PBS as a negative control. The LD_50_ at 35 days post infection was calculated using the previously established Bliss method [44].

Based on the LD_50_ result, 32 mice were randomized to four groups, each group of 4 groups, each of which contained four male mice and four female mice. On days 0 and 14, 16 mice were vaccinated with the 0.2 ml of liquid culture containing 1 × 10^7^ CFU of Δ*sseK2* mutant, seven days later, eight mice were orally inoculated with 0.2 ml of liquid culture containing 1 × 10^7^ CFU (lethal dose) of the WT and eight mice received 0.2 ml PBS. At the same time, 16 mice were orally inoculated with 0.2 ml PBS; seven days later, eight of these mice received 0.2 ml of liquid culture containing 1 × 10^7^ CFU (lethal dose) of the WT strain, and eight received 0.2 ml PBS.

### Bacterial load analysis

Forty-eight mice were divided randomly into three groups, each of which contained 15 mice. Three of the mice received an intraperitoneal injection of 0.2 ml PBS as a negative control. The other mice were infected with 1 × 10^5^ CFU of the different strains by intraperitoneal injection and were then killed at different time points (4 h, 24 h, 48 h, 72 h and 120 h). The spleen, liver, and PPs were collected from each mouse and homogenized in PBS. All samples were then serially diluted and evenly spread onto SS agar. The number of CFUs for each sample was determined 24 h later. For this animal experiment, the mice were anaesthetized with a 20% urethane (ethyl carbamate) solution. All animals were humanely handled.

### Statistical analysis

The data are showed as mean ± standard deviation (SD) and are representative of three independent experiments. One-way ANOVA was employed to identify the significant differences between the groups using the GraphPad Prism software version 5.0. A value of *P* < 0.05 was considered significant.

## Additional file

Additional file 1: ARRIVE guidelines. (PDF 191 kb)

## Data Availability

The data generated and/or analyzed during the current study are available from the corresponding author on reasonable request.

## Abbreviations

Cmr: Chloramphenicol
PCR: Polymerase chain reaction
SD: Standard deviation
